# The major vault protein is dispensable for zebrafish organ regeneration

**DOI:** 10.1016/j.heliyon.2020.e05422

**Published:** 2020-11-03

**Authors:** Xue Zhang, Yuxi Yang, Xiaoxue Bu, Yuanyuan Wei, Xin Lou

**Affiliations:** Medical School, Nanjing University, China

**Keywords:** Major vault protein, Regeneration, Zebrafish, Knockout, Anti-apoptotic effect, Biological sciences, Cell biology, Cell death, Developmental genetics, Regenerative medicine

## Abstract

As the main constituent of the largest cellular ribonucleoprotein complex, the evolutionary highly conserved major vault protein (MVP) has been proposed play vital roles in the regeneration of multiple organs. In current study, we use a *mvp* knockout zebrafish line recently generated to characterize the function of MVP during organ regeneration. We found the regenerative capacity of heart, spinal cord and fin is preserved in *mvp* knockout zebrafish. Further experiments demonstrated in injured *mvp* knockout zebrafish, the cell death is enhanced while the transcriptome landscape is largely unchanged. These data showed MVP acts as an anti-apoptotic factor at early phase of injury response while plays a dispensable role in the regenerative programs in zebrafish.

## 1 Introduction

The evolutionary highly conserved major vault protein (MVP) is the main constituent of the largest naturally occurring ribonucleoparticles known as vaults (Berger et al., 2009). Crystallography results shows 78 copies of the MVP protein assemble into the structural shell of vault (Tanaka and Tsukihara, 2012). Recently, studies have demonstrated that MVP is implicated in the regulation of multiple cellular processes including nucleocytoplasmic transport, signaling transduction, cellular differentiation, cell survival, and immune responses (Li et al., 2019; Minaguchi et al., 2006; Ryu et al., 2008; Vollmar et al., 2009). Due to majority of the research was carried out on *in vitro* systems, physiological role of MVP largely remains unknown.

Though an absolute function of cellular vaults has not been determined, several reports suggested these ribonucleoprotein particles might play a role in organ regeneration of zebrafish. Expression analysis revealed after injury, expression of MVP is induced in heart, caudal fin and spinal cord of zebrafish (Ma et al., 2018; Pan et al., 2013; Yoshinari et al., 2009). By using an morpholino-based knock-down approach, researchers observed reduced axonal regrowth after spinal cord transection and proposed MVP supports locomotor recovery and axonal regrowth in adult zebrafish (Pan et al., 2013). While so far the function of MVP on regeneration has not been systematically examined and evaluated by using genetic tools.

In this report, we generated a *mvp* knockout zebrafish line to characterize the function of MVP during organ regeneration. The *mvp*^*−/−*^ zebrafish are viable and show no obvious physical abnormalities. Our data indicate the absence of MVP leads no significant change on the regenerative capacities of heart, spinal cord and fin in zebrafish. Further experiments demonstrated in injured *mvp* knockout zebrafish, the cell death is significantly enhanced while the transcriptome landscape is largely unaltered. These data showed MVP acts as an anti-apoptotic factor at early phase of injury response while plays dispensable role in the regenerative programs in zebrafish.

## 2 Results

### 2.1 Expression of *mvp* during regeneration

**Figure 1:**
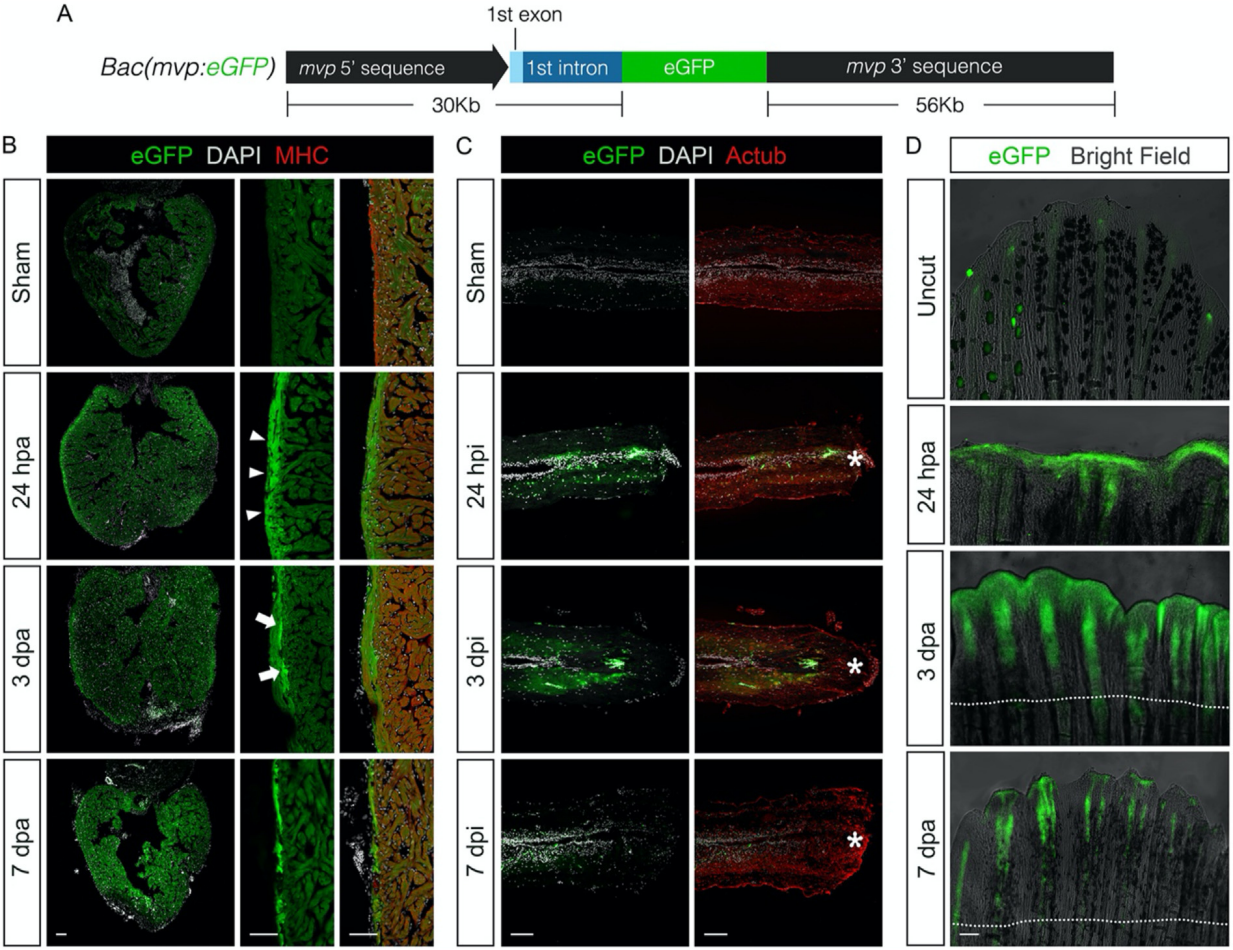
Expression analysis of *mvp* during zebrafish organ regeneration. (A) Schematic illustration of transgenic constructs for *mvp* reporter strain. (B–D) *mvp:EGFP* expression in heart, spinal cord and fin at designated time points. Arrowheads in B indicate expression in compact layer of ventricle wall. Arrows in B indicate expression in primordial layer of myocardium. Asterisks in C indicate injury epicenter. Dotted line in D indicate the approximate resection plane. A to C, scale bar = 100 μm.

To better define the spatiotemporal expression pattern of *mvp* during regeneration, we generated a *mvp:EGFP* reporter strain with BAC sequences containing zebrafish *mvp* (Figures 1A and S1) and evaluated EGFP signal in injured heart, spinal cord and fin. After resection of the ventricular apex, *mvp-*driven EGFP was induced throughout the outer compact layer of ventricular myocardium by 24 h. From 3 to 7 days post-amputation (dpa), the transactivation of EGFP was confined into primordial layer of myocardium then gradually switched off (Figure 1B and data not shown). By 24 h post spinal cord transection surgery, EGFP signals could be observed in the ependymal cells lining the central canal in adult zebrafish. As the regeneration process started, the EGFP expression briefly expanded to the neurons close to injure site and mostly disappeared at 7 days post-injury (dpi) (Figure 1C). After caudal fin amputation surgery, bright EGFP signal emerged in wound epidermis from about 24 h. During the formation of blastema, *mvp-*driven EGFP was expressed in the blastemal mesenchyme. At the regenerative outgrowth stage, EGFP signals was restrained into newly-formed actinotrichia, the non-mineralized spicules at the distal margin of the fin (Figure 1D). Altogether, these data showed the expression of *mvp* responses to the injure signals at acute response phase so MVP possibly plays a role in regulation of inflammation and cell survival during the early stage of regeneration in multiple organs.

### 2.2 Generation of *mvp* knockout zebrafish

**Figure 2:**
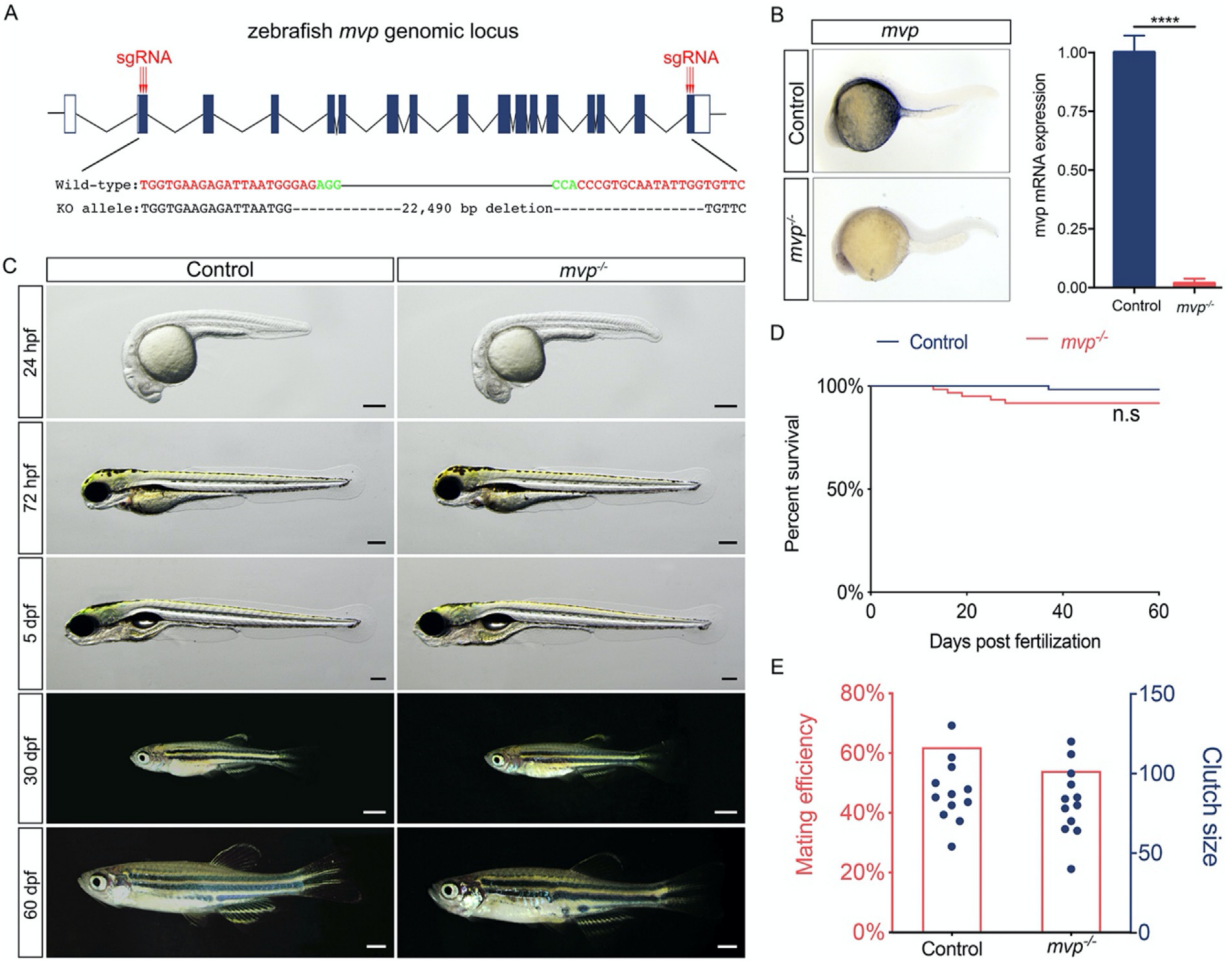
Generation of *mvp* knockout zebrafish line. (A) The zebrafish *mvp* genomic locus and Cas9/sgRNA targeting site. Deletions in Δ22,490 allele are shown as dashes. (B) *In situ* hybridization and qPCR results showing the absence of *mvp* mRNA in *mvp* knock out embryos. Data are mean ± SEM. ∗∗∗∗P < 0.0001. (C) Live images of control and *mvp^−/−^* zebrafish at designated time points. Lateral view, anterior to the left. For embryos and larvae, scale bar = 200 μm. For animal from 30 dpf to 60 dpf, scale bar = 2 mm. (D) Representative Kaplan-Meier plot for *mvp^−/−^* fish and clutchmates from one of three independent experiments. In each experiment, 60 *mvp^−/−^* animals and 60 total siblings were followed. n.s, not significant, Mantel-Cox test. (E) Fecundity (measured by clutch size) and mating efficiency (measured by percentage of fish laid embryos) in control and *mvp^−/−^* zebrafish. 30 *mvp^−/−^* fish and 30 age-matched control fish out-crossed with age-matched wild-type fish.

To elucidate the function of *mvp* in regeneration, we used CRISPR-Cas9 strategy to disrupt the zebrafish *mvp* gene. Multiple sgRNAs targeting *mvp* locus were designed (Figure 2A), transcribed and injected into 1-cell stage zebrafish embryo with Cas9 protein. F1 individuals were generated by cross the injected founders with wild-type AB fish and screened for presence of deletion of the whole *mvp* coding region. Among the alleles were obtained and displayed consistent phenotype (data not show) through this procedure, the one bearing a 22,490-bp deletion on *mvp* locus was chosen to be used in the subsequent analysis (Figure 2A and Figure S1). To confirm that loss of *mvp* in the knockout line, expression of *mvp* was determined by qPCR and whole mount *in situ* hybridization, the absence of *mvp* mRNA in *mvp*^*−/−*^ embryos was verified (Figure 2B). Embryos from multiple *mvp*^*+/-*^ incrosses were collected and raised to the adult stage to investigate the roles of *mvp* on zebrafish development, survival and fecundity. No perceivable morphological difference between *mvp*^*−/−*^ and wild-type animal was observed (Figure 2C). Similar to *mvp* knockout mouse (Mossink et al., 2003; Mossink et al., 2002), no significant effects on overall survival and fecundity was observed in *mvp*^*−/−*^ fish (Figure 2 D and E), so the *mvp* mutant fish presented an opportunity for investigating its function on regeneration.

### 2.3 The effect of loss of *mvp* on organ regeneration

**Figure 3:**
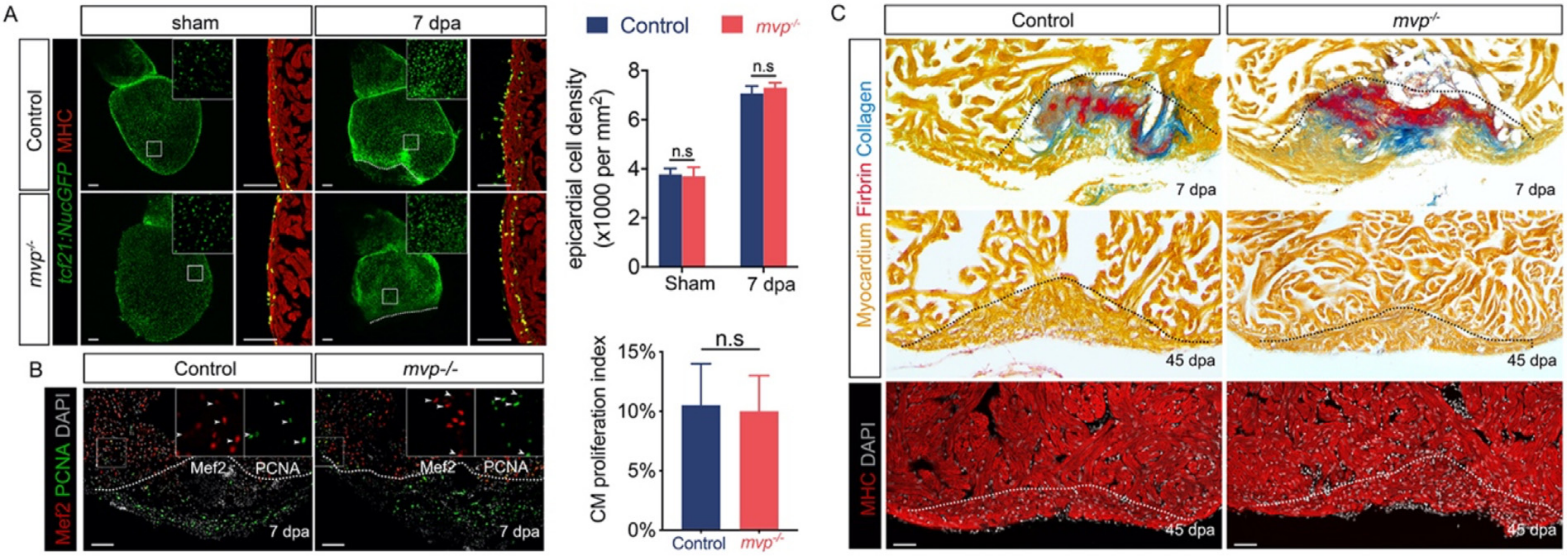
Heart regeneration in *mvp* KO fish. (A) Visualization of epicardial cells in control and *mvp^−/−^* fish. Epicardial cells proliferate by 7 dpi and are incorporated into the regenerating myocardial wall. Inset shows enlarged view of boxed area. Bar graph showing the density of epicardial cell at surface of hearts from control and *mvp^−/−^* fish. Data are mean ± SEM. For each group, eight samples were assessed. n.s, not significant. (B) Cardiomyocyte proliferation at 7 days post-amputation (dpa) in ventricular sections from control and *mvp^−/−^* animals, assessed by Mef2 and PCNA staining. Arrowheads, the PCNA positive. Scale bar = 100 μm. Bar graph showing cardiomyocyte proliferation indices on 7 dpa in control (n = 8) and *mvp^−/−^* (n = 8) fish. Proliferation data were collected for 4–6 sections per heart and averaged to generate each data point. Data are mean ± SEM. n.s, not significant. (C) Section images of ventricles at designated time points, assessed for myocardium (MHC) recovery and scar tissue (fibrin, collagen) removal. In A, B and C, dashed line indicates approximate resection plane.

The effect of loss of *mvp* on heart regeneration was analysed in ventricle resection fish, the most commonly used model in research (Gemberling et al., 2013). After surgery, the zebrafish heart carries out an organ-wide injury response, the epicardium get activated and play important structural and signaling roles for myocardium regrowth (Cao and Poss, 2018; Wang et al., 2015). The behavior of epicardial cells were visualized by using *tcf21:nucEGFP* transgenic line, the results revealed in both control and *mvp*^*−/−*^ animal, epicardial cells surrounding the ventricle were stimulated by injury to proliferate and became incorporated into the underlying myocardial wall by 7 day post-amputation (dpa) (Figure 3A). To determine whether loss of *mvp* affects the capacity of the zebrafish heart to regenerate, we examined the cardiomyocyte proliferation, scar tissue removal and muscle recovery. In all of the cases, no significant difference was observed between *mvp*^*−/−*^ and wild type animal (Figure 3 B and C). These results indicated the regenerative capacity of heart is unaltered in *mvp*^*−/−*^ fish.

**Figure 4:**
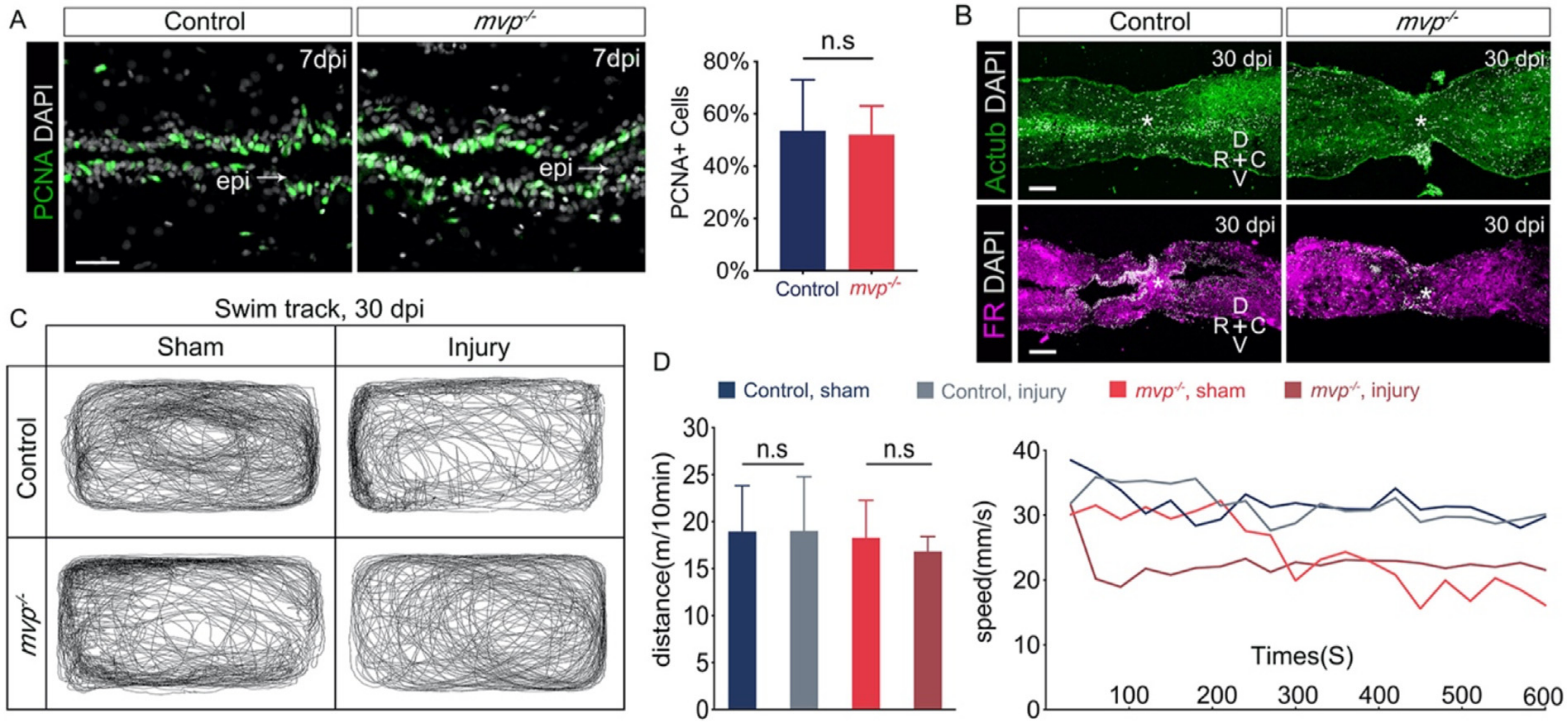
Spinal cord regeneration in *mvp* KO fish. (A) Proliferation of neural cells in control and *mvp^−/−^* animals on 7 day post-injury (dpi), assessed by PCNA staining. Bar graph showing percentage of proliferating neural cells in control (n = 6) and *mvp^−/−^* (n = 6) fish. epi, injury epicenter. (B) Upper panel, spinal cord structure of transected site by 30 dpi. Lower panel, retrograde tracing of axonal projections using Fluoro-Ruby (FR). Regenerating axons spanned the transection injury in both control fish (6/6) and *mvp^−/−^* fish (4/5; p < 0.05). Asterisks indicate injury epicenter. In A and B, scale bars = 100 um. (C) Representative swim tracking of individual animals. (D) Quantification of total distance (mean ± SEM, n = 6 for each group) and speed in (C). n.s, not significant.

It has been reported knock-down of MVP in zebrafish by morpholino resulted in reduced regrowth of axons after spinal cord injury (Pan et al., 2013). To examine whether *mvp* influence central nervous system regeneration, we investigated the axonal regrowth and locomotor recovery in zebrafish spinal cord transection model. In zebrafish, central nervous system regeneration occurs via *de novo* neurogenesis following the activation and proliferation of neural progenitor cells (Barbosa et al., 2015; Kroehne et al., 2011). To test whether MVP was required for regenerative cell proliferation, we quantified cell proliferation in the injured spinal cord and no significant difference was observed between *mvp*^*−/−*^ and wild type animal (Figure 4A). Using acetylated α-tubulin immunohistochemistry, we observed regenerating axons spanned at the transected site in the vast majority of wild-type and *mvp*^*−/−*^ samples by 30 day post-injury (dpi) (Figure 4B). By using retrograde tracing of axonal projections, reconnection of rostral and caudal stumps of spinal cords in both type of samples was verified (Figure 4B). Locomotor activity of fish was assessed as an index of functional recovery, the date indicated both wide type control and *mvp*^*−/−*^ fish had regained pre-injury swimming performance at 30 dpi (Figure 4 C and D). These results showed the absence of MVP leads no significant effect on zebrafish spinal cord regeneration.

**Figure 5:**
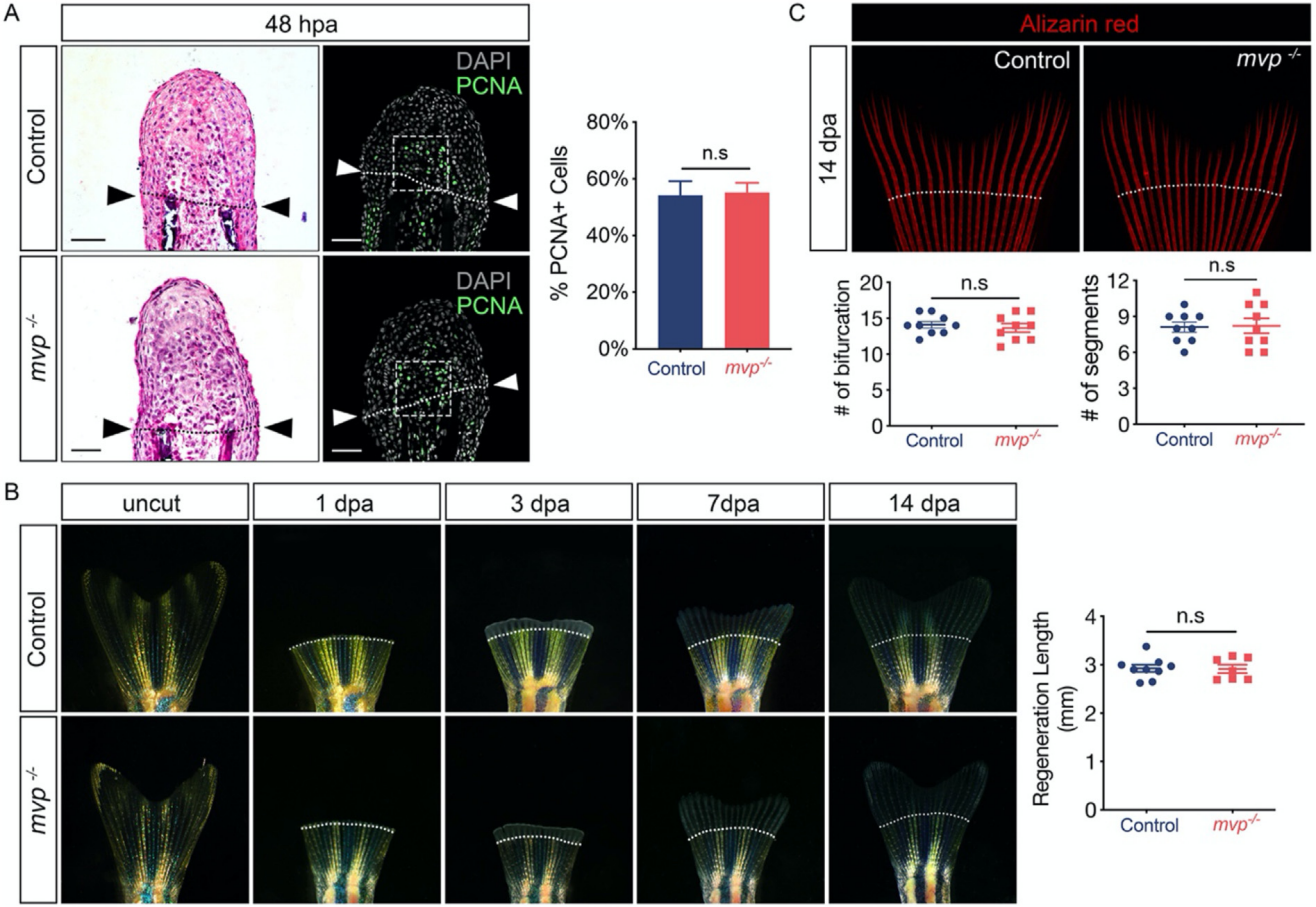
Fin regeneration in *mvp* KO fish. (A) Left panel, representative hematoxylin-stained sections of caudal fin regenerates (blastemal region) at 48 h post-amputation (dpa). Arrowheads indicate the plane of amputation. Middle panel, blastemal proliferation revealed by PCNA staining. Scale bar = 50 μm. Bar graph, cell proliferation (PCNA + cells) quantified in the blastema. PCNA + cell number was averaged among all sections spanning the entire fin width, and normalized to DAPI counts in the image. n.s, not significant. (B) Live images of fin regeneration in control and *mvp^−/−^* zebrafish at designated time points. Scatter plot with mean ± SEM to show the length of regenerated tissue at 14 dpa (n = 9 for each group). (C) Representative images of bone structure (revealed by Alizarin red staining) of caudal fin in control and *mvp^−/−^* fish on 14 dpa. Left lower panel, scatter plot with mean ± SEM to show the bifurcations in regenerated tissue (n = 9 for each group). Right lower panel, scatter plot with mean ± SEM to show the segment number in regenerated tissue (n = 9 for each group). n.s, not significant. In B and C, dashed line indicates approximate resection plane.

Then we examined the key processes of fin regeneration in *mvp*^*−/−*^ zebrafish. At 1–3 day post amputation, a mass of highly proliferative lineage-restricted mesenchymal progenitor cells, known as regeneration blastema, will formed at the injury site (Pfefferli and Jazwinska, 2015). H&E stained section shows loss of *mvp* did not significantly affect gross blastema morphology and size (Figure 5A). In *mvp*^*−/−*^ animal, cell proliferation in the mesenchymal region was comparable to wild type (Figure 5A). To further examine the fin regenerative capacity of in *mvp*^*−/−*^ fish, we amputated caudal fins from wild-type and *mvp*^*−/−*^ fish and continuously monitored the extent of new fin tissue growth for 14 days. The *mvp*^*−/−*^ fish showed similar regeneration rates to wild-type (Figure 5B). The zebrafish fins display elaborately radial pattern along the body axis, we examined possible function of *mvp* on bone patterning. Quantitative image analysis at 14 dpa revealed that *mvp*^*−/−*^ fish exhibited a comparable average number of segments and branching events (as measured by the number of bifurcations) in the regenerated ray (Figure 5C). Taken together, these data indicate the regenerative capacity of fin is unimpaired in *mvp*^*−/−*^ fish.

### 2.4 The changes of cell death and transcriptome in *mvp* knockout zebrafish

**Figure 6:**
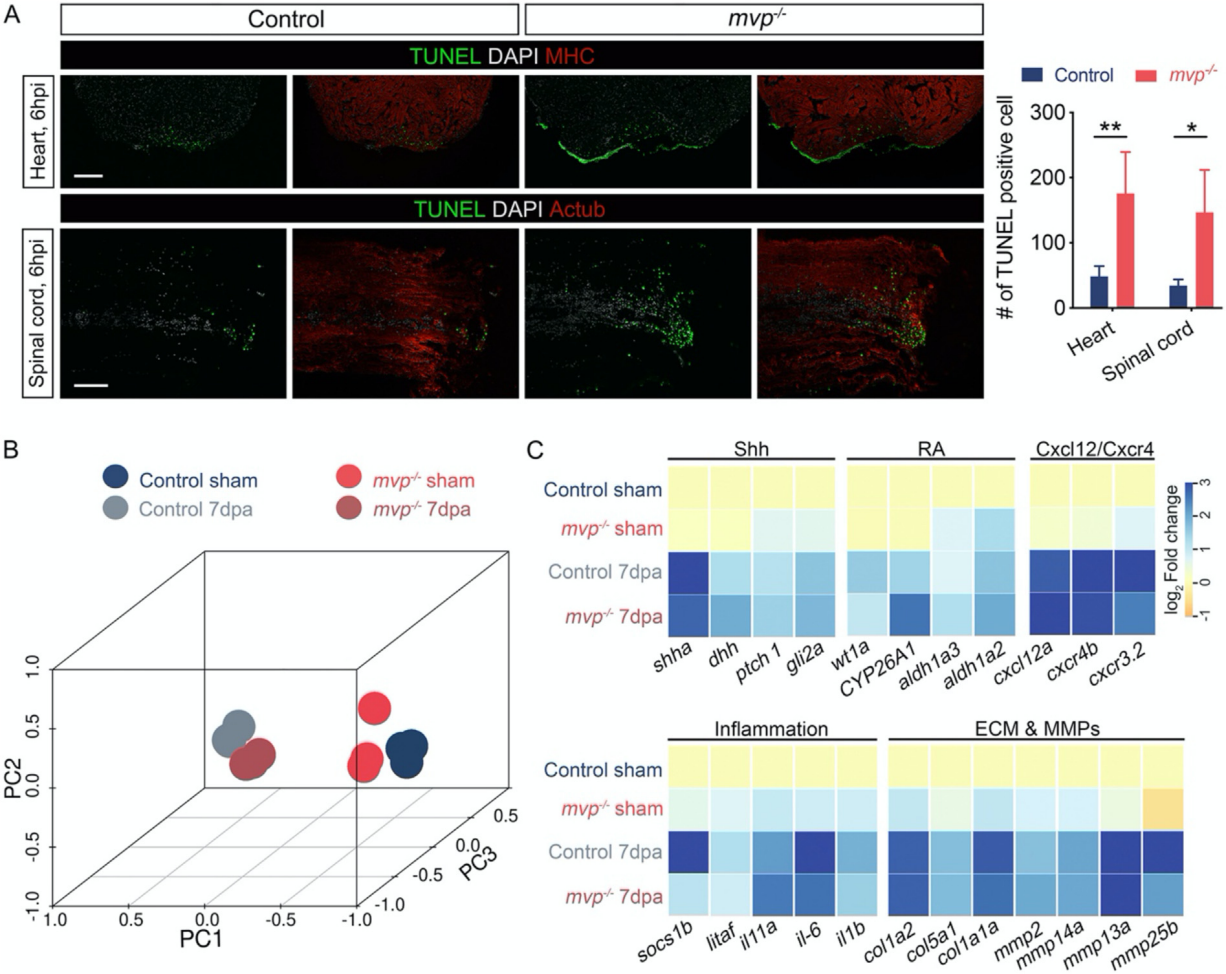
The effects of loss of *mvp* on injury-induced cell dearth and transcriptome landscape. (A) At 6 h post-surgery, cell death in heart and spinal cord was assessed with TUNEL staining. Bar graph showing number of TUNEL + cells, data are mean ± SEM. ∗∗P < 0.001, ∗P < 0.05. Data were collected for 6–8 sections per experiment set. Scale bar = 100 μm. (B) Principal component analysis (PCA) plots from the whole transcriptome of hearts from control and *mvp^−/−^* fish. (C) Heat maps of selected gene sets. The colour intensity represents the log2 fold change of the gene expression.

As in human, injury results in rapid local tissue necrosis and triggers apoptosis in zebrafish (Gonzalez-Rosa et al., 2011; Hui et al., 2010; Saraste et al., 1997). Evidence is accumulating that MVP is implicated in cell survival by modulating the expression of apoptosis regulator genes such as Bcl-2 (Berger et al., 2009; Ryu et al., 2008). We therefore examined apoptosis in injured heart and spinal cord, TUNEL assay revealed in both type of tissues, cell death was significantly enhanced in *mvp*^*−/−*^ fish (Figure 6A).

Our previous results indicated the regenerative capacity is preserved in *mvp* knockout zebrafish, it interesting to check whether the regenerative programs are altered, so we analyzed the change on transcriptome landscape in regenerating hearts of wild-type and *mvp*^*−/−*^ animal by RNA-seq. PCA analysis showed the transcriptome profiles were closely clustered based on status of heart regeneration, suggesting that the response to regenerative signal in *mvp*^*−/−*^ animal are similar to wild-type (Figure 6B). Zebrafish heart regeneration is a highly dynamic process and multiple signals and factors have been reported involved in it to composing a complex regenerative program (Gonzalez-Rosa et al., 2017), we manually examined the expression of these regeneration-related genes and found majority of them showed comparable level of transactivation in *mvp*^*−/−*^ animal compare to wild-type (selected gene sets were plotted in Figure 6C). Collectively, these data indicate MVP acts as an anti-apoptotic factor at early phase of injury response and loss of MVP has no oblivious effect on regenerative program of heart.

## 3 Discussion

In this study, we have successfully generated a MVP knockout zebrafish line with CRISPR/Cas9 technology. The entire coding region of MVP (about 22kb) was deleted, *mvp* mRNA is undetectable in knockout animals so the allele should act as a true null allele. Apparently, as in mammalian, vaults do not play an essential role in the development of teleost and in cellular processes in a normal environment. Our data further indicated vaults are involved in a mechanism needed for cell survival under the injury-induced cellular stress. This result is corroborative with the observation that *mvp* was transactivated at the acute phase of injury response in all three types of tissues analysed. Through extensive cell death was noticed in *mvp* knockout zebrafish, no long term effect on regeneration was detected. On possible explanation for this observation is the excessive loss of cell was fully replenished ascribe to the extraordinary regeneration capacity of zebrafish. It is interesting to examine the effect of loss of *mvp* on organ regeneration/tissue repair in animals with limited regeneration capacity. Since MVP knockout mice are viable, this type of experiments is feasible and would be informative.

Beside MVP, vaults consist of two other proteins, TEP1 and VPARP, as well as one non-coding RNA (vRNA) (Berger et al., 2009). Interestingly, none of these three vault components was transactivated after injure in zebrafish (data not shown). One of the possibilities is the pre-existing protein/RNA was recruited to form the injury-induced vaults, the alternative is MVP alone give rise to vault-like particles, like what has been noticed in insect cells (Stephen et al., 2001). It would be interesting to investigate the actually composition of vaults under different biological circumstances.

It has been reported in zebrafish, MVP acts as a negative regulator of IFN production by restricting the activation of TBK1(Li et al., 2019). Our transcriptome data showed the intensity of inflammation was not significantly altered in injured *mvp* knockout fish (Figure 6C). More systematic analysis need be performed to clarify the role of *mvp* on immune response at various situations.

## 4 Methods

### 4.1 Animals

All animal experimentations were carried out in accordance with approved guidelines of the Institutional Animal Care and Use Committee of the Nanjing University. All zebrafish lines were kept on a AB background.

### 4.2 Generation of *BAC(mvp:EGFP)* zebrafish

The translational start codon of mvp gene in the BAC clone CH211–152F6 was replaced with a EGFP cassette using Red/ET recombineering technology (GeneBridges). The recombined BAC was linearized by I-SceI digestion, purified DNA was co-injected with meganuclease into one-cell stage zebrafish embryos. The full name of this transgenic line is *Tg(mvp:EGFP)*^*nju211*^.

### 4.3 Generation of mvp knockout zebrafish

gRNAs were designed by CRISPR/Cas9 target online predictor (https://cctop.cos.uni-heidelberg.de). 50 pg of sgRNAs and 500 pg of Cas9 protein were co-injected into one-cell stage embryos. Mvp Δ49,190 allele was identified and used for subsequent experiments. Genotyping oligos used are: mvp KO allele: 5′ GTTTTATTTAGGCTAGAATAAAAGCAGCGTCT 3′ and 5′ GCAGCATAAAACACAAAGCTCTTTGGGTTCGAC 3′; mvp WT allele#1: 5′ GTTTTATTTAGGCTAGAATAAAAGCAGCGTCT 3′ and 5′ CCCGGCAACAAAGAAAACAGGATATAC 3′; mvp WT allele#2: 5′ GGTATGTCTAACCTGACCTGTGTTTTAAATCC 3′ and 5′ CAACAGCCGCCTTTACTCTGATTTACTGAGTG 3′.

### 4.4 Histochemistry

Immunofluorescence was done as previously described (Sarmah et al., 2010).

In brief, tissues were dissected and fixed in 2% paraformaldehyde (PFA) in Phosphate buffer saline (PBS) overnight at room temperature for 2h, immersed in Tris-HCl(150mM, pH9.0) at 70 °C for antigen retrieval, embedded in OCT and frozen for cryosection on a Leica cryostat (Leica, CM3050S). 10 μm cryosections were used in all experiments. Sections were blocked with goat serum and primary antibodies were incubated overnight at 4 °C. Secondary antibodies linked to appropriate fluor were incubated for 2 h at room temperature. Sections were mounted with Vectashield medium. The primary antibodies used in this study were: mouse anti-TNNT2 at 1:50 (Santa Cruz, sc-20025); mouse anti-Ace Tubulin at 1:40 (sigma, T6793); mouse anti-PCNA at 1:200 (Santa Cruz, sc-56); rabbit anti-Mef2 at 1:50 (AnaSpec, AS-55609). The secondary antibodies used in this study at a concentration of 1:500 were: Anti-mouse IgG (H + L), F (ab')2 Fragment (Alexa Fluor® 594 Conjugate) (CST, 8890s); donkey-anti-Mouse IgG 488 (ThermoFisher, A21202); donkey-anti-mouse IgG 568 (ThermoFisher, A10037); donkey-anti-rabbit IgG 568 (ThermoFisher, A10042). DAPI (Roche) was used at a concentration 1:1000.

Cardiomyocyte proliferation indices were quantified by manually counting Mef2+ and Mef2+/PCNA + cells in injury regions. To determine the proliferation index of each heart, data from 4–6 sections with the largest ventricular wound area were averaged.

Acid Fuchsin-Orange G (AFOG) staining was performed on paraffin heart sections (13 um) as described (Poss et al., 2002). Paraffin sections were deparaffinized with 3 rounds of xylene followed by rehydration with serial dilutions of ethanol baths prior to staining. The slides were transferred into preheated Bouins fixative (Sigma, HT10132) and incubated for 2 h at 60 °C and another hour at room temperature. The slides were washed twice for 20 min in tap water and then incubated in 1% phosphomolybdic acid for 5 min. After rinsing with distilled water, sections were incubated for 5 min with AFOG staining solution [1g aniline blue diammonium salt (Sigma, 415049); 2g Orange G (Sigma, O7252) in 100 ml acidified distilled water] and again washed with distilled water. The sections were dehydrated in 95% ethanol and twice in 100% ethanol for 2 min. After passing through three consecutive xylene baths, the slides were mounted with Neutral Balsam Mounting Medium (Sangon Biotech, E675007).

### 4.5 Quantitative real-time PCR

Total RNA was prepared using TRIzol (Invitrogen, 15596) and Direct-zolTM RNA Miniprep (Zymo Research, R2052) from control and mvp mutant samples. cDNA was synthesized with PrimeScript RT kit (Takara, RR047A). RT-qPCR reactions were performed on the Poche LightCycler system using the SYBR Green Master Mix (Takara, RR420A). Melt curves were examined to ensure primer specificity. Primers used in RT–qPCR were designed to span exon-exon junctions and were listed above: #1: 5′ AAGGTTGGAGCGTATCTGCC 3′ and 5′ GCACGTGAAGGGCTTTCTTG 3′; #2: 5′ AACGGGAAAGTCGGGCTAAG 3′ and 5′ CACGCACCTTTCCGGTTTTG 3′.

### 4.6 Surgery procedures

Heart surgery was performed on zebrafish anesthetized in Tricaine as described (Wang and Poss, 2016). Iris scissors was used to make an incision in the skin above the heart and the silver-colored pericardial sac was exposed. Tweezers was used to gently pull up the apex of the ventricle. Remove approximately 20% of the ventricular apex with vannas scissors. Once the wound has clotted and stopped bleeding, the fish can be returned to a tank with aquarium water. Use a pipette to gently push water across the gills of the fish until it is able to swim freely on its own (generally 3–5 min).

Spinal cord injury was performed on zebrafish anesthetized in Tricaine as described (Becker et al., 1997). Iridectomy scissors were used to make a longitudinal incision through the skin at approximately 3.5 mm rostral to the anterior border of the dorsal fin, which corresponds to 15/16^th^ vertebrate. The spinal cord was exposed and completely transected with scissors. A cotton swabs was used to clean the blood and surrounding muscle tissues were place back to cover the vertebral wound. Tissue glue was used to reduce the wound size. The fish was returned to a tank with aquarium water and gently push water over the gills so that the fish can recover from anaesthesia quickly. Fishes were allowed to regenerate for a specified period of time at 28 °C for follow-up analysis.

Fin surgery was performed on zebrafish anesthetized in Tricaine as described (Pfefferli and Jazwinska, 2015). The fish was placed on the petri dish under a dissecting microscope. Blunt forceps was used to lift the tail fin from the dish and iridectomy scissors was used to make a single vertical cut that is perpendicular to the rays of the fin. one-half of the caudal fin was amputated and the fish was returned to the tank until recovery.

### 4.7 FR dye staining

Iridectomy scissors were used to make an incision through the skin at approximately 3–4 mm caudal to the spinal cord transection site. The spinal cord was exposed, and dry crystals of 1 μl of Fluoro-Ruby (tetra-methyl rhodamine dextran, 10,000 MW; Thermo Fisher Scientific, D1816) were placed on the surface of the exposed spinal cord. The incision was closed, and the spinal cord was collected 24 h after the dye insertion for histological analysis.

### 4.8 Swimming track assay

A swim-tracking test was performed as previously described (Fang et al., 2014). Zebrafish were placed in an opaque acrylic tank (length: 11.5 cm; width: 9 cm) containing aquarium water (depth: 6 cm) and acclimatized for 2 min. Zebrafish swimming was recorded for 10 min with Ethovision XT software (Noldus, Wageningen, Netherlands). The number of zebrafish analyzed for this assay was eight for each group. Uninjured and sham-injured zebrafish were used as controls.

### 4.9 Alizarin red staining

For whole-mount Alizarin Red staining, 4% PFA-fixed fins were rehydrated through a decreasing methanol series, bleached for 30 min in 1% KOH, 1.5% H2O2. Subsequently, fins were washed twice with water and washed for 20 min in a saturated Alizarin Red solution containing 1% KOH, followed by several washes with water and transferred to glycerol for imaging.

#### 4.9.1 Hematoxylin-eosin staining

For hematoxylin-eosin staining, 4% PFA-fixed fin were hydrated through an increasing methanol series and embedded in paraffin. It was sectioned in 5um. The sections were de-paraffinised in 2 changes of xylene and rehydrated in a decreasing methanol series. The sections were stained in Harris hematoxylin solution for 15 min and differentiated in 1% acid alcohol for 30 s. After hydration, the sections were stained in eosin-phloxine solution for 30 s. The slides were mounted with Neutral Balsam Mounting Medium (Sangon Biotech, E675007) after xylene cleaning.

### 4.10 TUNEL assay

For TUNEL reactions, the staining with anti-digoxigenin fluorescein conjugated was performed according by In Situ Cell Death Detection Kit (Sigma, 11684795910). The cryosections were post-fixed for 20 min in 4% PFA, washed twice 10 min in PBS and permeated in 0.1% sodium citrate with 0.1% Triton-X. After washing in PBS, DNA breaks were elongated with Terminal Transferase and Digoxigenin-dUTP solution. After a wash in PBS, the sections were used for immunohistochemistry as described above.

### 4.11 RNA in situ hybridization

Transcription of DIG-labeled antisense RNA probes was performed using standard methods. Whole-mount RNA in situ hybridization (WISH) was carried out as previously described (Thisse and Thisse, 2008).

### 4.12 Imaging

Whole imaging was performed using a Leica DFC320 camera on a Leica M205FA stereomicroscope. All confocal images were acquired using a Zeiss LSM880 confocal microscope.

### 4.13 Survival curve

Sixty each WT siblings and mvp^−/-^ animals were put into 3 L tank from 7 dpf. Every three days, the number of living fish was counted till 60 dpf. Kaplan- Meier curve was generated with Prism 6 (GraphPad) (Goel et al., 2010).

### 4.14 RNA sequencing

RNA was extracted from 9 hearts of wild type and mvp^−/−^ fish by using the Direct-zol RNA prep Kit (ZYMO Research, Cat. No. 2052). The libraries were constructed with NEBNext® Single Cell/Low Input RNA Library Prep Kit for Illumina (NEB, Cat No.6420) and sequenced on illumina Novaseq platform. HISAT2 V2.1.0 was used to map the sample sequencing reads to the GRCz11 reference genome. Gene expression counts were calculated using FeatureCounts v1.6.0. based on current Ensemble annotation. The R package DESeq2 was then employed to make differential gene expression calls. RNAseq data have been deposited in NCBI's Gene Expression Omnibus and can be accessed through GEO Series accession number GSE157170.

### 4.15 Statistical analysis

Statistics were performed using GraphPad Prism 7 Software. Survival curve was analyzed with Mantel-Cox tests. Other statistical tests were performed using two-sided, unpaired Student's t-tests and where numerical data are presented as mean ± SEM. Differences were considered significant if the probability value was P < 0.05 and highly significant if the probability value was P < 0.01. All experiments were carried out with at least three biological replicates. The numbers of animals used are described in the corresponding figure legends.

## Declarations

### Author contribution statement

Xue Zhang: Performed the experiments.

Yuxi Yang: Analyzed and interpreted the data.

Xiaoxue Bu, Yuanyuan Wei: Contributed reagents, materials, analysis tools or data.

Xin Lou: Conceived and designed the experiments; Analyzed and interpreted the data; Wrote the paper.

### Funding statement

Xin Lou was supported by National Natural Science Foundation of China (NSFC 31671505 and 31970765).

### Competing interest statement

The authors declare no conflict of interest.

### Additional information

Data associated with this study has been deposited at NCBI's Gene Expression Omnibus (GEO) under the accession number GEO: GSE145495.

Supplementary content related to this article has been published online at https://doi.org/10.1016/j.heliyon.2020.e05422.
