# Supplementary material for: The major vault protein is dispensable for zebrafish organ regeneration
Source: Heliyon. 2020 Nov 3;6(11):e05422. doi: 10.1016/j.heliyon.2020.e05422 (PMC7644919; doi:10.1016/j.heliyon.2020.e05422)
Supplement: Supplementary file 1 — Supporting Information [file mmc1.pdf]

Supplemental Information

The Major Vault Protein is Dispensable for Zebrafish Organ Regeneration

Xue Zhang[1], Yuxi Yang[1], Xiaoxue Bu[1], Yuanyuan Wei[1], Xin Lou[1, *]

1, Model Animal Research Centre, Nanjing University, China.

Contents:

1. Sanger sequencing result for mvp knockout.
2. EGFP expression pattern of *BAC(mvp:EGFP)* during embryogenesis.

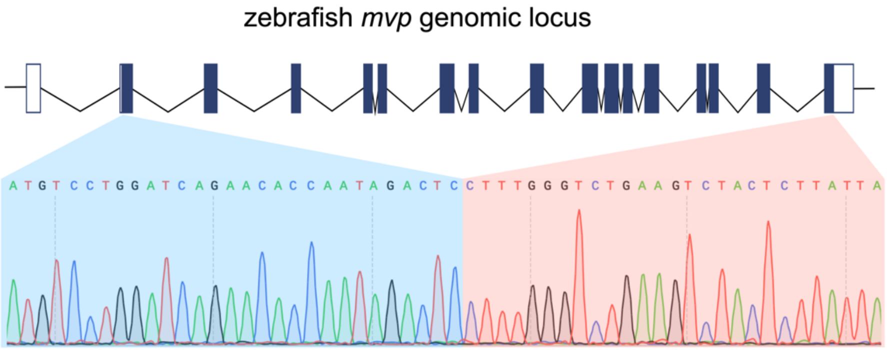

Supplemental Figure 1. Sanger sequencing of genotyping PCR products indicating the 22,490 bp deletion caused by the Cas9 protein and gRNA.

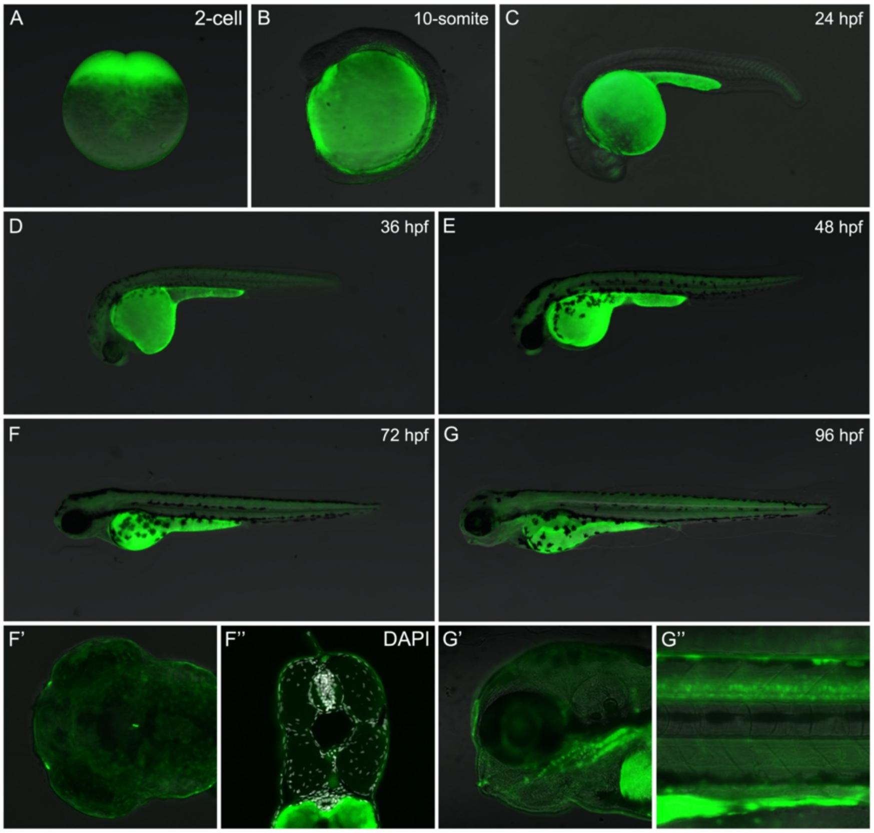

Supplemental Figure 2. EGFP expression pattern of *mvp:EGFP* transgenic line during embryogenesis. EGFP signal could be observed in the 2-cell stage embryo from female transgenic fish, indicated the maternal deposition of *mvp* (A). From 10-somite stage, zygotic expressed EGFP could be observed in yolk syncytial layer (B to G). As development proceeded, the EGFP signal was gradually emerged in pituitary gland (F'), skin (F''), olfactory placodes (G'), esophagus (G') and neurons in spinal cord (G''). B, lateral view with anterior to the top. C to G, lateral views with head to left. J and L, ventral views with anterior to the top. F', ventral view. F'', cross section of trunk. G' and G'', lateral view.
